# Review of the assessment and management of neonatal abstinence syndrome

**DOI:** 10.1186/1940-0640-9-19

**Published:** 2014-09-09

**Authors:** Sarah Mary Bagley, Elisha M Wachman, Erica Holland, Susan B Brogly

**Affiliations:** 1 Section of General Internal Medicine, Boston University School of Medicine, 801 Mass Ave. 2nd Floor, Boston, MA 02118, USA; 2 Department of Pediatrics, Boston Medical Center, 771 Albany Street, Dowling 4N 4109, Boston, MA 02118, USA; 3 Department of Obstetrics and Gynecology, Boston Medical Center, 85 East Concord Street, 6th Floor, Boston, MA 02118, USA; 4 Department of Epidemiology, Boston University School of Public Health, 715 Albany Street, Talbot Building East, Boston, MA 02115, USA

**Keywords:** Neonatal abstinence syndrome assessment, Neonatal abstinence syndrome management, Neonatal abstinence syndrome treatment

## Abstract

Neonatal abstinence syndrome (NAS) secondary to in-utero opioid exposure is an increasing problem. Variability in assessment and treatment of NAS has been attributed to the lack of high-quality evidence to guide management of exposed neonates. This systematic review examines available evidence for NAS assessment tools, nonpharmacologic interventions, and pharmacologic management of opioid-exposed infants. There is limited data on the inter-observer reliability of NAS assessment tools due to lack of a standardized approach. In addition, most scales were developed prior to the prevalent use of prescribed prenatal concomitant medications, which can complicate NAS assessment. Nonpharmacologic interventions, particularly breastfeeding, may decrease NAS severity. Opioid medications such as morphine or methadone are recommended as first-line therapy, with phenobarbital or clonidine as second-line adjunctive therapy. Further research is needed to determine best practices for assessment, nonpharmacologic intervention, and pharmacologic management of infants with NAS in order to improve outcomes.

## Introduction

Between 2000 and 2009, antepartum opioid use increased from 1.19 to 5.63 per 1000 live births in the United States. Concurrently, the incidence of neonatal abstinence syndrome (NAS) increased from 1.20 to 3.39 per 1000 live births, and related hospitalization costs increased from $39,400 to $53,400 per infant with NAS [1]. NAS is characterized by gastrointestinal, respiratory, autonomic, and central nervous system disturbances from opioid withdrawal that affect critical regulatory areas of postnatal life adaptation [2]. Fifty to eighty percent of opioid-exposed infants require pharmacologic treatment for NAS [3-5]. Recently, studies have suggested that in-utero exposure to nicotine, selective serotonin reuptake inhibitors, and benzodiazepines may influence NAS [6,7]. A better understanding of the effects of maternal opioid–agonist medications, breastfeeding, genetic factors, and polypharmacy on the severity of withdrawal and the need for pharmacotherapy will help to optimize assessment and management [3,6,8,9].

Despite this growing problem, great variability persists in the assessment and management of opioid-exposed infants, with only half of neonatal intensive care units (NICUs) with accredited fellowships having a protocol for NAS management [10]. In addition, no medications are currently approved by the FDA for use in NAS management or prenatal opioid dependence. Buprenorphine and methadone are currently labeled as FDA Category C: *“Animal reproduction studies have shown an adverse effect on the fetus and there are no adequate and well-controlled studies in humans, but potential benefits may warrant use of the drug in pregnant women despite potential risks.”* It is important to recognize that although these drugs may not be FDA approved for use in pregnancy, the risks associated with continued use of illicit drugs or misuse of prescription medications is far greater. In addition to the benefits of methadone or buprenorphine to treat withdrawal symptoms and reduce cravings, pregnant women in treatment may have access to other treatment such as counseling, infection screening, and fetal growth monitoring, which can lead to improved pregnancy and neonatal outcomes [11]. There are decades of experience with methadone use in pregnancy, and it has long been considered the gold standard in treating pregnant women with opioid dependence. Methadone has been shown to decrease intrauterine growth restriction, prematurity, and fetal distress compared to heroin use only [11-14]. Recently, the World Health Organization included a recommendation for women with opioid dependence to be maintained on either methadone or buprenorphine during pregnancy [15]. While some studies suggest that prenatal buprenorphine decreases NAS severity compared to methadone, the interpretation of these comparative studies is limited by important differences in women treated with buprenorphine versus methadone, such as bias from study dropout and confounding by indication [3,16-21]. Nonetheless, it is important that women have access to two effective treatment options and that both have reasonable safety profiles for the exposed fetus.

Given this variability in clinical practice, several clinical narrative reviews have been written to guide practice for NAS management, focusing primarily on pharmacologic treatment regimens [4,5,22,23]. Those reviews have commented on the lack of randomized trials or solid evidence guiding current assessment and management of NAS. The objective of this review is to summarize available evidence on the assessment and management of infants exposed to opioids in utero, including assessment tools used for NAS scoring, nonpharmacologic interventions, and pharmacologic management of NAS.

## Methods

We conducted a systematic search of the published English-language literature of studies on 1) assessment of NAS, 2) nonpharmacologic interventions for NAS, and 3) pharmacologic treatment of NAS. To identify articles, two of the authors (SMB and EH) searched PubMed and the Cochrane Database and searched the references in identified articles. Articles published between 1975 and November 15, 2013, are included in the results.

For assessment articles, the search terms included were: *neonatal abstinence syndrome assessment, neonatal abstinence score, neonatal drug withdrawal score, neonatal drug withdrawal assessment, Finnegan score, Lipsitz score,* and *modified Finnegan score*. For the nonpharmacologic intervention articles, we used the search terms: *neonatal abstinence syndrome nonpharmacologic treatment, neonatal abstinence syndrome management,* and *neonatal abstinence syndrome treatment.* For pharmacologic management, the search terms were: *neonatal abstinence syndrome pharmacologic treatment*, *neonatal abstinence syndrome management,* and *neonatal abstinence syndrome treatment*.

Different inclusion criteria were applied for the three study categories due to varying availability of data and types of studies conducted in each area. We included studies of assessment tools developed for the clinical diagnosis and management of NAS. For nonpharmacologic treatment, we included studies composed of cohort, case series, case–control, and randomized controlled trials (RCTs). For pharmacologic treatment, only RCTs and nonrandomized experimental studies were included. For nonpharmacologic intervention and pharmacologic treatment studies, articles were only included if they defined objective NAS outcome measures. We excluded studies that examined the use of paregoric as pharmacologic treatment because although it was commonly used in NAS treatment in the past, it is no longer recommended due to issues with impurity [4,24]. NAS outcomes measures extracted included inter-rater variability for NAS scoring, length of pharmacologic treatment for NAS, peak NAS scores, mean NAS scores, and length of hospital stay.

## Results

### Assessment

Of 368 articles identified through our search terms, eight specifically pertained to the evaluation of assessment tools for NAS. Of those eight articles, four are included in this review. In addition, we have included the results of the assessment tool used in the MOTHER (Maternal Opioid Treatment: Human Experimental Research) study. The four excluded studies were eliminated due to clinical issues. For example, one excluded study used infant sucking as the study outcome of NAS improvement; however, routine use of this measurement tool is not practical for most nurseries because it requires an instrument to measure sucking, which is not practical, and whose use is uncommon [25]. A second excluded study evaluated neurobehavioral assessment using the NICU Network Neurobehavioral Scale, which was not meant to be used in routine clinical care for the treatment of withdrawal in infants with NAS [26]. A third excluded study used an adjuvant tool whose use is not intended to replace a full assessment tool [27]. The fourth excluded study examined the use of an actigraph (portable motion detector that measures movement), which would not be practical for clinical use [28].

The assessment tools for NAS are provided in Table 1. They were all developed in the United States. The first was the Neonatal Abstinence Syndrome Score (NASS), or what is commonly referred to as the Finnegan Score (FS). This scale or a modified version is the most widely used NAS scale in the US [3,5]. It had a high inter-rater reliability coefficient of 0.82 when initially developed [29,30]. The second tool developed was the Narcotic Withdrawal Score, or the Lipsitz Score [31]. There was no inter-rater reliability determined; only infants who scored above a certain threshold had been exposed to opioids in utero*,* and that score was established as the threshold score for NAS treatment. The next scale developed is called the Neonatal Narcotic Withdrawal Index, which has an inter-rater reliability coefficient of r = 0.771 (p < 0.01) [32]. The fourth scale we reviewed is the Neonatal Withdrawal Inventory (NWI). The NWI was able to correctly identify withdrawing infants with a specificity and sensitivity of 100 percent when Finnegan’s NASS was used as the gold standard. The inter-rater reliability coefficient was 0.89–0.98 for the NWI, compared with 0.7–0.88 for Finnegan’s NASS [33]. The final scale is the MOTHER NAS scale, which is a modified Finnegan scale. They estimated an intraclass correlation coefficient for the degree of agreement between an expert rater and a site rater (the MOTHER trial was an international, multisite trial) and found that the lowest coefficient exceeded 0.94 [3].

**Table 1:** Studies of tools to assess neonatal abstinence syndrome

**Reference**	**Index vs. reference group(s)**	**Scale**	**Primary results**
**Finnegan, 1975**[29]	37 infants assessed with NASS vs. 37 infants born prior to development of NASS	Neonatal Narcotic Abstinence Scoring System	Mean inter-rater reliability coefficient: 0.82 (0.75–0.96)
	Exposure methadone and heroin	20 items, weighted on pathologic severity	Management of NAS without drug Rx: 30% vs. 46%
			LOS: 6 days vs. 8 days
			Total Rx days decreased by 25%
**Lipsitz, 1975**[31]	41 infants evaluated by 2 pediatric residents, placed in 5 groups, one group which was opioid exposed	Narcotic Withdrawal Score	Only the infants exposed to opioids had scores ≥ 5; concluded that a score > 4 suggests a clinical threshold for Rx
	Exposure methadone and heroin	11 items, scored 0–3 based on severity	
**Green, 1981**[32]	Infants exposed to methadone or heroin(n = 50)	Neonatal Narcotic Withdrawal Index	Inter-rater reliability coefficient: 0.771*
	Control infants (n = 40)	7 items, scored 0–2 based on severity	Mean NAS score on day 2 of life: 1.57 vs. 3.08*
		7th item is “other” and includes 12 other symptoms	
**Zahorodny, 1998**[33]	Group A: opioid-exposed infants with NAS (n = 30)	Neonatal Withdrawal Inventory	Group A: inter-rater reliability coefficient: 0.89–0.98
	Group B: opioid infants with NAS (n = 12) and nonopioid-exposed controls (n = 13)	8 items given predetermined weights	Group B: sensitivity and specificity for NAS diagnosis: 100%, 100%
	Group C: opioid-exposed infants with NAS (n = 25)		Group C: sensitivity and specificity for Rx threshold vs. the NASS: 100%, 100%
**Jones, 2010**[3]	131 opioid-exposed infants, scored using the same scale	MOTHER NAS scale (modified Finnegan)	Intraclass correlation coefficient > 0.94
	Exposure methadone or buprenorphine		

*Abbreviations:NAS* Neonatal Abstinence Syndrome, *NASS* Neonatal Abstinence Scoring System, *Rx* Treatment, *LOS* Length of Hospital Stay.

*indicated p < 0.05.

### Nonpharmacologic interventions

Of the 879 articles we identified pertaining to nonpharmacologic interventions, 13 were included in this review.

#### *Infant feeding method*

The majority of evidence for the nonpharmacological management of NAS is in the area of infant feeding method. Breastfeeding is recommended in stable mothers on methadone and buprenorphine maintenance therapy who are not concurrently using illicit drugs. The definition of stable will vary by institution; for example, the policy may state that in order to be eligible to breastfeed, the mother will have had no positive urine toxicology screens and been adherent with treatment in the last trimester [11,22,34,35]. Transfer of methadone and buprenorphine into breast milk is minimal and unrelated to maternal dose [8,36]. Breastfeeding has been shown to act as an analgesic for infants and is established as beneficial for soothing agitated infants [37].

Seven studies were identified that examined the role of infant feeding method on NAS outcomes [9,38-43]. All studies were retrospective cohorts of primarily full-term infants exposed to methadone, heroin, and buprenorphine and were determined to have NAS symptoms. Studies did not consistently differentiate between expressed breast milk and breastfeeding. Some studies did not compare exclusive breastfeeding versus combination feeding and those fed with formula only. Duration of breastfeeding was also not defined by the majority of studies. Five of the seven studies specified the NAS scoring assessment used, but there was variation both in the threshold for initiating pharmacologic treatment and the type of pharmacologic treatment used. Criteria for permitting breastfeeding were not described in every study, which introduced bias that was not addressed in these studies.

The primary findings of the seven studies are presented in Table 2, indicating an overall decreased need for pharmacologic treatment, a decrease in NAS scores, and decreased length of pharmacologic therapy and hospitalization for infants who were breastfed primarily or breastfed to any extent. In four of the studies, length of hospitalization was 3–19 days shorter in breastfed infants [9,38,41,43]. In four of the studies, infants who were breastfed to any extent had up to 30 percent reduction in their need for pharmacologic treatment for NAS [9,38,41,42]. In addition, one study found that predominantly breastfed infants demonstrated signs of withdrawal significantly later than formula-fed infants (10 vs. 3 days; p < 0.001), with decreased Finnegan withdrawal scores in the first 9 days of life [38].

**Table 2:** Studies of the nonpharmacologic management of neonatal management syndrome

**Reference**	**Study design**	**Study objective**	**Index vs. reference group(s)**	**Assessment tool**	**Results**
**Abdel-Latif, 2006**[38]	Retrospective Cohort	To determine association between breastfeeding and NAS outcomes	Breastfed infants with NAS (n = 85)	Finnegan Score	Rx for NAS: OR = 0.36 (CI 0.18–0.71)*
			Formula-fed infants with NAS (n = 105)		Mean LOS: 14.7 (SD 14.9) vs. 19.1 (SD 15.0) days*
**Dryden, 2009**[42]	Retrospective Cohort	To determine association between breastfeeding and NAS outcomes	Breastfed infants with NAS (n = 99)	Modified Lipsitz	Rx for NAS: OR = 0.55 (CI 0.34–0.88)*
			Formula-fed infants with NAS (n = 351)		
**McQueen, 2011**[39]	Retrospective Cohort	To determine association between breastfeeding and NAS outcomes	Breastfed infants with NAS (n = 8)	Modified Finnegan	Mean # of NAS scores: 25.0 (SD 23.5) vs. 56.2 (SD 39.1) vs. 95.6 (SD 34.6)*
			Combination-fed infants with NAS (n = 11)		Mean NAS score 4.9 (SD 2.9) vs. 6.5 (SD 3.7) vs. 6.9 (SD 4.2)*
			Formula-fed infants with NAS (n = 9)		
**Pritham, 2012**[43]	Retrospective Cohort	To determine association between breastfeeding and NAS outcomes	Breastfed with NAS (n = 14)	Not specified	Mean LOS: -3.3 (SE 1.7) days
			Infants combination-fed with NAS (n = 22)		
			Formula-fed infants with NAS (n = 96)		
**O’Connor, 2013**[40]	Case Series	To describe association between breastfeeding and NAS outcomes	Breastfed infants with NAS (n = 65)	Modified Finnegan	Mean NAS score: 8.83 (SD 3.56) vs. 9.65 (SD 2.58)
			Formula-fed infants with NAS (n = 20)		Rx for NAS: 23.1% vs. 30.0%
**Wachman, 2013**[9]	Prospective Cohort	To determine association of genetic variables with NAS outcomes	Breastfed infants with NAS (n = 38)	Modified Finnegan	Rx for NAS: 50% vs. 77%*
			Formula-fed infants with NAS (n = 48)		Mean LOS: 15.8 (CI 11.5–20.1) vs. 27.4 (CI 22.5–32.3) days*
**Welle-Strand, 2013**[41]	Ambi-directional Cohort	To determine association between breastfeeding and NAS	Breastfed infants with NAS (n = 95)	Modified Finnegan	Rx for NAS: 53% vs. 80%*
		outcomes	Formula-fed infants with NAS (n = 29)		Mean length of Rx: 28.6 (SD 19.1) vs. 46.7 (SD 26.3) days*
**Hunseler, 2013**[44]	Retrospective Cohort	To describe association between rooming-in and NAS outcomes	NAS infants exposed to rooming-in (n = 24)	Finnegan	Finnegan Score > 12: 6.3% vs. 6.4%
			Control NAS infants (n = 53)		Rx for NAS: 79.2% vs. 88.7%
					Mean length of Rx: 27 vs. 32.5 days
					Mean LOS: 38 vs. 41.5 days
**Abrahams, 2007**[45]	Retrospective Cohort	To describe association between rooming-in and NAS outcomes	Infants with NAS exposed to rooming-in (n = 32)	Modified Finnegan	Mean length of Rx: 5.9 vs. 18.6 days*; 5.9 vs. 18.6 days*
			Control infants with NAS (n = 38) (historical comparison)		Mean LOS: 11.8 vs. 23.5 days; 11.8 vs. 25.9 days*
			Control infants with NAS (n = 36) (from another institution)		
**D’Apolito, 1999**[46]	Nonblinded Random Assignment of Rx	To determine the association between infants sleeping in a rocking bed vs. controls with NAS outcomes	Infants with NAS exposed to rocking bed (n = 7)	Finnegan	Mean NAS score on day 7 of intervention: 10.2 (SD 2.1) vs. 8.0 (SD 1.8)
			Control infants with NAS (n = 7)		
**Oro, 1988**[47]	Randomized Assignment of Rx with Matched Controls	To determine the association between infants sleeping in a waterbed vs. controls and NAS outcomes	Infants with NAS exposed to a waterbed (n = 15)	Finnegan	Maximum NAS score: 6.2 (SD 0.7) vs. 6.4 (SD 1.0)
			Control infants with NAS (n = 15)		Mean LOS: 10.5 (SD 1.2) vs. 11.5 (SD 3.4) days
**Maichuk, 1999**[48]	Randomized to Intervention; Nurses Blinded to Hypothesis	To determine the association between sleeping position with NAS outcomes	Infants with NAS placed in prone position (n = 25)	Finnegan	Maximum NAS score: 10.52 (SD 2.08) vs. 13.17 (SD 2.03)*
			Infants with NAS placed in the supine position (n = 23)		Mean NAS score: 5.11 (SD 0.64) vs. 7.60 (SD 0.70)*
**Filippelli, 2012**[49]	Case Series	To describe the possible effects of NIA on infants with NAS	Infants with NAS exposed to NIA (n = 54)	Not applicable	Chart review revealed improvements in calming, sleep, and feeding

*Abbreviations:NAS* Neonatal Abstinence Syndrome, *Rx* Treatment, # Number, *LOS* Length of Hospital Stay, *OR* Odds ratio, *CI* 95% confidence interval, *SD* Standard deviation, *SE* Standard error.

*p < 0.05.

#### *Other nonpharmacologic interventions*

Other nonpharmacologic interventions that have been described include rooming-in, bed type, positioning of the infant, and non-insertive acupuncture (NIA). The primary findings are presented in Table 2. Two retrospective studies of rooming-in found overall decreased length of hospital stay and duration of therapy [44,45]. Two studies examined the role of bed type on NAS outcomes. In the first study, infants randomly assigned to a rocking bed with accompanying intrauterine sounds had higher mean withdrawal scores that were statistically nonsignificant and an increase in sleep disturbance that was only statistically nonsignificant on day 7 of life [46]. In the second study, in which infants were randomly assigned to an experimental nonoscillating waterbed or a regular crib, those in the waterbed required less pharmacologic treatment for NAS [47]. In another study on positioning, 48 infants were randomly assigned to either supine or prone sleeping positions; those infants placed in a supine position had significantly higher peak and mean withdrawal scores and higher mean caloric intake [48]. Finally, one study examined the role of NIA on NAS as adjunctive therapy for infants receiving treatment for NAS. Infants included in the study were diagnosed although their pharmacologic treatment was not specified. After the NIA sessions, babies were subjectively noted to have improved sleep, with decreased restlessness and improved feeding [49].

### Pharmacologic management

Out of 940 published articles identified using our search terms for pharmacologic treatment, seven studies met our inclusion criteria. Of note, there are studies included in the Cochrane Reviews that are not included in our review because they examined studies using paregoric or unpublished data. The primary results of the seven selected studies are presented in Table 3.

**Table 3:** Studies of pharmacologic treatment for neonatal abstinence syndrome

**Reference**	**Design**	**Purpose**	**Comparison (Index vs. Reference)**	**Assessment measure**	**Primary outcome**	**Results**
**Jackson, 2004**[50]	Double-blind RCT	Efficacy of opioid vs. phenobarbitone as first-line therapy	Morphine (n = 41) Phenobarbitone (n = 34)	Lipsitz	Mean length of Rx	8 vs. 12 days* (unadjusted)
**Langenfeld, 2005**[51]	Randomized Trial, Blinding Not Specified	Compare tincture of opium to oral morphine	Morphine (n = 17)	Finnegan	Mean length of Rx	29.8 vs. 26.9 days
			Tincture of Opium (n = 16)		Mean LOS	37.5 vs. 32.4 days
**Kraft, 2008**[52]	Phase I, Randomized, Open Label, Active Control	Feasibility and safety of buprenorphine in Rx of NAS	Buprenorphine (n = 13)	Modified Finnegan	Mean length of Rx	22 (SD 12) vs. 32 (SD 16) days
			Neonatal opium solution (n = 13)		Mean LOS	27 (SD 11) vs. 38 (SD 13) days
**Kraft, 2011**[53]	Phase I, Randomized, Open Label, Active Control	Feasibility and safety of buprenorphine	Buprenorphine (n = 12)	Modified Finnegan	Mean length of Rx	23 vs. 38 days*
			Morphine (n = 12)		Mean LOS	23 vs. 42 days
**Coyle, 2002**[54]	Partially Randomized, Controlled Trial	Assess whether Rx with DTO + phenobarbital vs. DTO alone is better	DTO + phenobarbital (n = 10)	Finnegan	Finnegan Scores	Infants in placebo group, spent more time with FS > 7 *
			DTO + placebo (n = 10)			Infants in phenobarbital group spent more time with FS < 5*
					Mean LOS	Mean: 38 vs. 79 days*
**Agthe, 2009**[55]	Randomized, Double-Blinded Controlled Trial	To assess use of clonidine as an adjunct therapy to opioids to manage NAS	Clonidine (n = 40)	Modified Finnegan	Mean length of Rx	11 (95% CI 8–15) vs. 15 (95% CI 12–17) days
			Placebo (n = 40)			
**Surran, 2013**[56]	Randomized, Nonblinded Controlled Trial	Clonidine versus phenobarbital to reduce # days of Rx with morphine sulfate	Phenobarbital (n = 34)	Modified Finnegan	Length of Rx with morphine sulfate	B = -4.6 days (95% CI 0.3–8.9)*
			Clonidine (n = 32)			

*Abbreviations:NAS* Neonatal Abstinence Syndrome, *Rx* Treatment, # Number, *LOS* Length of Hospital Stay, *DTO* Diluted Tincture of Opium.

*indicated p < 0.05.

Two studies compared morphine with phenobarbitone and with diluted tincture of opium (DTO), respectively. In the first study, 75 infants were enrolled in a double-blind RCT to compare morphine with phenobarbitone in infants with two consecutive Lipsitz scores of > 4. Median treatment duration was significantly shorter in the morphine group. Infants treated with phenobarbitone required more frequent second-line treatment with chloral hydrate (47% vs. 35%; p = 0.11) and admission to a specialty care baby unit (62% vs. 30%; p = 0.04) [50]. The second study compared randomized treatment of NAS with tincture of opium versus morphine in 33 infants and used the FS assessment for NAS treatment initiation and discontinuation. Both length of treatment and length of stay were longer in the morphine group (29.8 vs. 26.9 days and 37.5 vs. 32.4 days; p > 0.05) [51].

Two studies examined the use of buprenorphine to treat NAS. In both studies, a modified FS was used to assess infants, and three consecutive scores of ≥ 24 was the threshold for treatment. The first study enrolled 26 infants to a Phase I, randomized, open label, active-control study. The active-control arm therapy was neonatal opium solution. Although designed as a safety study, the mean length of treatment for the buprenorphine group (22 vs. 32 days) and of hospital stay (27 vs. 38 days) was clinically but not statistically significant [52]. In the second study of a revised dosing schedule based on these safety data, statistically significant differences were seen in length of treatment and length of stay (Table 3) [53].

Three studies examined the efficacy of an adjunct medication versus first-line therapy alone to reduce treatment duration and hospital stay for infants with NAS, all of which were RCTs (Table 3). Adjunct medication with clonidine (two studies) and phenobarbital (one study) was started at the same time as the first-line opioid in all three studies. These studies showed that adjunct therapy could reduce the duration of treatment with first-line therapy at clinically important levels, resulting in an average cost savings of ~ $36,000, but that the duration of phenobarbital treatment after discharge could be long, with a range of 2–9 months. Finally, when clonidine was compared to phenobarbital as an adjunct medication, infants treated with phenobarbital had a shorter duration of treatment [54-56].

## Discussion

The initial development of NAS scoring systems in the 1970s was a crucial turning point in the care of infants exposed to opioids when pediatricians recognized the need for a consistent way to assess exposure. However, since that time, the most commonly used FS has been modified and is used differently across institutions. Nurseries and NICUs have highly variable practices that range from use of published abstinence tools to inconsistent assessment strategies [4,10,57]. As the incidence and clinical impact of NAS rises, it is critical for infant care that clinicians employ a common, objective, and validated tool to guide diagnosis and treatment of NAS*.* In other disease states and syndromes, there are agreed-upon criteria for diagnosis, and the same standards should be expected for infants with NAS. Without consistency, it is impossible to assure quality care. The American Academy of Pediatrics (AAP) recommends use of a standardized tool such as the gold-standard Finnegan abstinence assessment for evaluation of NAS [4]. They also recommend an inter-observer reliability program offered for quality improvement efforts. We recommend the use of such established educational programs utilizing the FS manual and DVD (such as that offered by Neo Advances) to standardize scoring among care providers [58]. This standardization may lead to a decrease in the extent of pharmacotherapy used and length of hospitalization needed for affected infants across the United States. The Lipsitz tool includes similar signs and symptoms, but is shorter. Despite the widespread use of these various tools, validation and inter-observer reliability data is lacking. Infants are typically scored using various tools every 3–4 hours starting shortly after birth and are monitored for 5–10 days for signs of opioid withdrawal in the hospital. If infants meet a threshold score on the assessment tool, such as > 8 on the commonly used FS, they are typically started on pharmacologic therapy. Morphine and methadone remain the two most commonly used first-line medications, with lack of evidence for which agent is superior. Morphine is given orally and is typically dosed every 3–4 hours at 0.3–1.0 mg/kg/day, titrated to effect, and then weaned every 24–48 hours. Methadone is also given orally and dosed every 4–12 hours, titrated in a similar range of 0.3 – 1.0mg/kg/day, and then weaned over time. Methadone-weaning protocols vary greatly, with some institutions weaning this medication as an outpatient treatment over a longer period of time. Administration of second-line agents for more severe withdrawal consists of a variety of agents including phenobarbital, clonidine, and clonazepam [4,5,24].

Although limited, the available evidence suggests that nonpharmacologic interventions may provide some benefit for infants with NAS by decreasing clinical symptoms and the need for pharmacologic therapy. Standard practice for treating drug-exposed infants includes limiting exposure to sounds and lights and promoting clustering of care, swaddling, and holding, as well as breastfeeding for eligible patients. Evidence is growing that breastfeeding is beneficial for this population [9,38-43]. Breastfeeding is recommended by AAP for the first 6 months of life for all infants without contraindications. Although specific criteria vary by institution, the general recommendation is that infants exposed to opioids in utero should be breastfed if the mother is enrolled in a substance abuse program. Even in those mothers who are eligible, there have generally been low breastfeeding rates in this population, likely secondary to inherent feeding difficulties in these infants [12,34,35,59]. Other interventions such as rooming-in may also be beneficial and cost effective. Both breastfeeding and rooming-in can provide opportunities for bonding and also normalize the postpartum process for women who may feel vulnerable and stigmatized because of their opioid use history. As it would be difficult to conduct randomized trials of interventions that are current standard of care, further studies should focus on how to increase the rates of breastfeeding and rooming-in. The limited available evidence about NIA suggests it may be beneficial, but more studies are needed and a randomized controlled design would be feasible.

There are currently no FDA-approved medications for NAS, and the data supporting one specific treatment is lacking. The studies examining pharmacotherapy efficacy are small; they have used different assessment tool and protocols to escalate and wean medications and were inconsistently adjusted for covariates such as maternal smoking history, exposure to other substances, and feeding methods and are therefore very difficult to compare. There is a need for high-quality RCTs to determine best practices and to establish safety and efficacy. The AAP recommends use of oral morphine solution or methadone when indicated, but notes that the best options for adjunct therapy are not known as well as the best treatment options for infants with polyexposure [4]. Although preliminary, the use of buprenorphine to treat NAS shows promise, but further research should be done that includes feasibility testing because administering it sublingually might present some administrative challenges.

The number of infants exposed to opioids in utero and developing NAS has dramatically risen in the last 10 years; based on current trends, the incidence will increase over the next decade. In addition, as knowledge about predicting NAS severity based on genetics and prenatal treatment of the mother expand, rigorous high-quality research should follow. As illustrated by the evidence for nonpharmacologic interventions, it is crucial to consider the infant with NAS as part of an infant–mother dyad because treatment of the infant does not occur in isolation from the mother. Creating a more secure, compassionate, and comfortable environment for the dyad will likely optimize outcomes for both mother and infant. Currently, the care for those infants, including assessment and management, varies widely across the country, and existing guidelines are based on minimal data. We recommend that nurseries adopt standard protocols that include use of published assessment tools and accepted NAS treatment such as morphine and methadone, as well as the training of all staff involved in infant care. Further research is urgently needed to assure the best care for infants with NAS, some of which should focus on the variations of NAS expression.

## Competing interests

The authors declare that they have no competing interests.

## Authors’ contributions

SMB and SBB were responsible for the conception and design. SMB and EH performed the literature search. SMB, EMW, and EH reviewed the articles. SMB, EW, EH, and SBB drafted the manuscript. All authors read and approved the final manuscript.
